# Structure Optimization of Planar Nanoscale Vacuum Channel Transistor

**DOI:** 10.3390/mi14020488

**Published:** 2023-02-19

**Authors:** Ji Xu, Congyuan Lin, Yu Li, Xueliang Zhao, Yongjiao Shi, Xiaobing Zhang

**Affiliations:** 1 School of Electronic and Information Engineering, Nanjing University of Information Science and Technology, Nanjing 210044, China; 2 Joint International Research Laboratory of Information Display and Visualization, School of Electronic Science and Engineering, Southeast University, Nanjing 210096, China

**Keywords:** nanoscale vacuum channel, field emission, fabrication

## Abstract

Due to its unique structure, discoveries in nanoscale vacuum channel transistors (NVCTs) have demonstrated novel vacuum nanoelectronics. In this paper, the structural parameters of planar-type NVCTs were simulated, which illustrated the influence of emitter tip morphology on emission performance. Based on simulations, we successfully fabricated back-gate and side-gate NVCTs, respectively. Furthermore, the electric properties of NVCTs were investigated, showing the potential to realize the high integration of vacuum transistors.

## 1. Introduction

Recently, nanoscale vacuum channel transistors (NVCTs) have attracted widespread attention from researchers due to their promising prospects in terms of fast response, RF, and high reliability [1,2,3,4,5]. In NVCTs, a vacuum is used as the medium for electron transport and metals are used as the materials for electron emission and collection. With greater resistance to irradiation than conventional semiconductors, they are widely used in space electronics. Writing in Nature Electronics, Jin-Woo Han and colleagues at NASA’s Ames Research Center and Glenn Research Center designed an NVCT fabricated with SiC and verified that the NVCT could withstand ionizing radiation, such as gamma rays and neutrons, substantially reducing the damaging effects of space radiation on silicon electronics. This is due to vacuum channels and metal emitters and collectors, both of which are inherently immune or less susceptible to radiation damage. NVCTs also perform better in high frequency environments, where carriers are ballistically transported in the vacuum channel, dramatically reducing transport times, increasing operating frequencies, and even promising increases to the THz band as new RF devices. Generally, device structures are classified into planar or vertical types, depending on the difference in vacuum channel morphology [6,7]. In previous reports, no dielectric layer between the gate and channel in vertical-type NVCTs were included, so the electric field in the channel could be effectively modulated by the gate [7,8,9,10,11]. However, it also leads to the gate current leakage problem, which cannot be fundamentally solved.

On the other hand, planar-type NVCTs with a typical back-gate structure are generally isolated by a dielectric layer from the gate and the nanoscale vacuum channel, which can greatly suppress the gate leakage current [12,13,14,15,16]. Moreover, the planar structure means that the emitter and the collector of the device are in the same plane, in which a sub-100 nm vacuum gap is created between them by high-precision processing [17]. Initially, the electrons are emitted from the emitter into the vacuum nano-gap, and transit to the collector in the form of ballistic transport [18,19], thus eventually creating the current. Therefore, the structural parameters of the emitter/collector electrodes, such as the emitter morphology and vacuum channel length, could extensively influence the emission current and operating voltage [20]. Furthermore, back-gate NVCTs are more compatible with existing IC processes, making them more attractive to researchers [21,22,23].

In this paper, we simulated and optimized the structural design of NVCTs, providing a theoretical basis for the actual device fabrication. Furthermore, planar-type NVCTs were fabricated by high-precision electron beam lithography (EBL) and a subsequent lift-off process. Finally, the corresponding electrical measurements were carried out to investigate the device’s field emission performance and operational stability, exploring its potential application prospects.

## 2. Materials and Methods

### 2.1. Simulation

The software used for the simulation was CST Studio Suite, and the material chosen for the simulation was Perfect Electric Conductor (PEC). The environment of the simulation was set in a complete vacuum, eliminating the influence of environmental factors on the simulation. The 3-D electrostatic and particle solver in CST was used to obtain the input and output characteristics of the NVCTs.

The grid should be set more densely at the edges of the device than at the center during simulation. In addition, this experiment analyzes the current and voltage of the emitter and collector. A denser grid needs to be inserted near the emitter and collector regions for higher sensitivity. An overly dense grid will consume computational memory, increasing computation time and reducing the efficiency of the simulation. Therefore, a balance between computational memory and simulation time is required when performing the meshing.

### 2.2. Fabrication of NVCT

**Figure 1:**
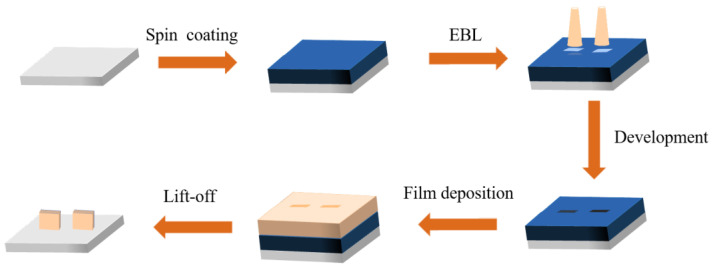
Schematic diagram of the fabrication process.

Based on the simulations, we fabricated the planar NVCT for subsequent performance measurements. The fabrication process includes conventional semiconductor processes, as shown in Figure 1. The fabrication process includes the spin coating of the photoresist, electron beam exposure, thin film deposition, and the lift-off process of the gold electrode. Firstly, the photolithographic layout is part of the design of the nano-vacuum trench structure, and we used direct write exposure in the study, which required a pre-determined exposure pattern recognizable to be drawn by L-edit graphic design software and converted into a data format recognizable by the exposure device. Subsequent to pre-processing the substrate, the substrates were placed in an oven for drying to remove any residual water vapor after cleaning. Secondly, the substrate was spin-coated with a PMMA film as the photoresist. A clean square substrate was placed on the vacuum chuck of the spin coater and an appropriate amount of PMMA photoresist was applied to the center of the substrate using a dropper. The substrate was baked on a heating table to fix the photoresist. Then, the PMMA film was exposed to an electron beam with an exposure dose of 600 μC/cm^2^. Then, the substrate was placed in a developer for 120 s, and then transferred to isopropanol for 60 s for fixing, and was finally blow dried using a nitrogen gun. After development, the Au film was deposited by the electron beam evaporation process. The sample with the gold electrode deposited was placed in an acetone solution for photoresist stripping. After stripping, the sample was washed in an isopropyl alcohol solution and blow dried with nitrogen gas. Finally, the sample was dried in an oven at 90 °C to complete the stripping. The final step was the lift-off process, followed by a post-annealing of the substrate. The post-treatment could remove the residual photoresist and improve the strength of the Au film.

### 2.3. Characterization

After the fabrication of the nano-vacuum channel structure was completed, the gold thin film electrode structure needed to be further analyzed and characterized to confirm whether the prepared structure significantly deviates from the structure designed for the device. In this paper, a FEI Quanta 200 Scanning Electron Microscope (SEM) was applied to characterize the microscopic morphology of the nanoscale vacuum channel structure. SEM characterization was used to observe whether the pattern size met the design specification, whether the electrode spacing was severely widened, whether the electrode shape was intact, and whether the photoresist was cleanly removed. In addition, due to the special characteristics of field emitters, the emitter side may be destroyed during testing due to transient high currents caused by surface impurities, uneven tips from the stripping process, etc. Therefore, if there is a significant degradation in performance, SEM characterization is also required to analyze the cause.

## 3. Results and Discussion

### 3.1. Optimization of Emitter Morphology

For emitters, the current density (j) at the emitting surface is positively related to the surface electric field strength (*E*), according to classical field emission theory [24,25]. The field emission current is positively related to the emission area (α), while the surface electric field strength (*E*) is positively related to the field enhancement factor (*β*), the collector voltage (*V_A_*), and the reciprocal of the vacuum channel length (1/*d*). These mechanisms provide the design directions for enhancing the field emission capability of the devices. For instance, the emission current in NVCTs is generated by the emitter, the magnitude of which is directly determined by the electric field strength at the emitting surface of the emitter electrode. The expression for the electric field strength (*E*) at the surface of the emitter is as follows.

(1) $$E=\beta \frac{V_{A}}{d}$$

The magnitude of the field enhancement factor (*β*) in the above expression depends on both the properties of the emitter material and the geometry emitter tip. In general, the aspect ratio of the emitter increases with decreasing tip dimensions, improving the field enhancement factor.

This feature is usually exploited by the reported cold cathode field emission devices. One outstanding feature of this design is that the electric field distribution can be tightly focused at the emitter tip. By designing the emitter in the form of a sharp cone, the radius of curvature is decreased, which correspondingly increases the field enhancement factor, thereby reducing the turn-on voltage and increasing the emission current [26]. Thus, the field emission capability of nanoscale vacuum channel structures can be improved by reducing the radius of curvature (r) of the emitter tip.

Combined with the numerical simulations, the performance of the device can be predicted to a certain extent. It is essential to simplify the structural model with an appropriate physical mechanism, which would affect the rationality of the calculation. Here, we optimize the emitter tip morphology to reduce the turn-on voltage and increase the emission current. The device model of the NVCT is based on the classical field emission as follows [24,25]:

(2) $$j_{FN}=AE^{2}exp\left(-\frac{B}{E}\right)$$

where *A* and *B* are fixed constants, *A* = 2.66 × 10^−11^ A/V^2^, *B* = 4 × 10^9^ V/m.

**Figure 2:**
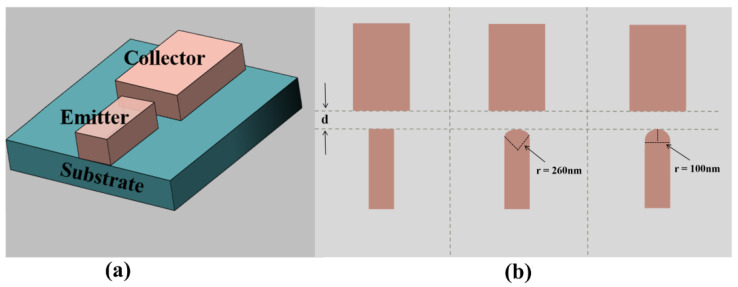
Schematic diagram of the structural model, (**a**) three-dimensional schematic, (**b**) top view of planar-type NVCTs with emitter of flattened electrode, radius of curvature 260 nm, and radius of curvature 100 nm.

**Figure 3:**
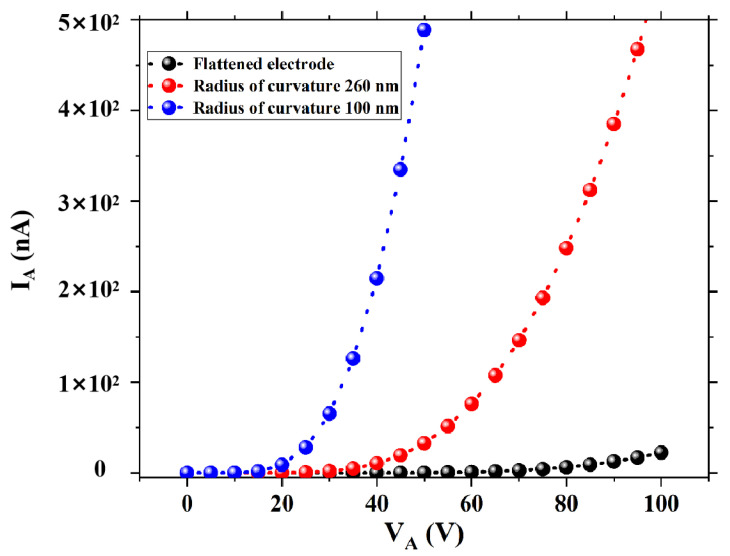
Emission current versus collector voltage for different emitter tip morphology.

To illustrate the influence of emitter tip morphology on field emission performance, three different types of planar-type NVCTs were designed. Figure 2 shows the top view of planar-type NVCTs with the emitter of the flattened electrode, which has a radius of curvature of 260 nm and 100 nm, respectively. The vacuum channel length d is fixed, set as 100 nm. In addition, the emission current versus collector voltage is shown in Figure 3, from which the turn-on voltage decreases from ~80 V to ~20 V, and the emission current increases by an order of magnitude with the decreasing radius of curvature.

### 3.2. Device Performance Measurement

**Figure 4:**
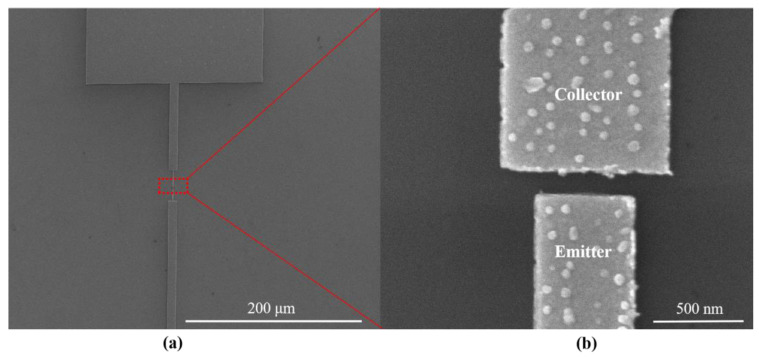
SEM images of (**a**) fabricated planar-type NVCT, and (**b**) the zoom-in of the nanoscale vacuum channel structure.

Further characterization of the devices was required after fabrication. As shown in Figure 4a, a planar-type NVCT with a flat emitter structure was successfully fabricated, with a vacuum channel of ~100 nm. Figure 4b shows the zoom-in of the nanoscale vacuum channel structure.

**Figure 5:**
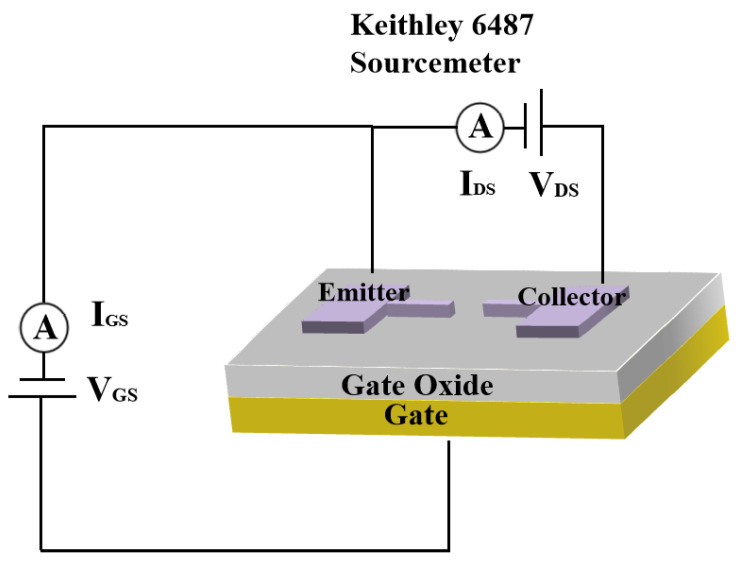
Schematic diagram of the test setup.

The schematic diagram of the test setup is shown in Figure 5, where the Keithley 6487 emitter meter connects the emitter to the collector, providing the emitter-collector voltage V_G_ and recording the emission current. The output characteristic curve is obtained by recording the emission current while scanning V_A_, with a fixed gate voltage V_G_. Furthermore, we can obtain a cluster of output characteristic curves by varying the V_G_. Furthermore, it is necessary to measure under vacuum conditions to avoid ionization. In the test, the vacuum level in the vacuum chamber is set at around 10^−4^ Pa at room temperature.

**Figure 6:**
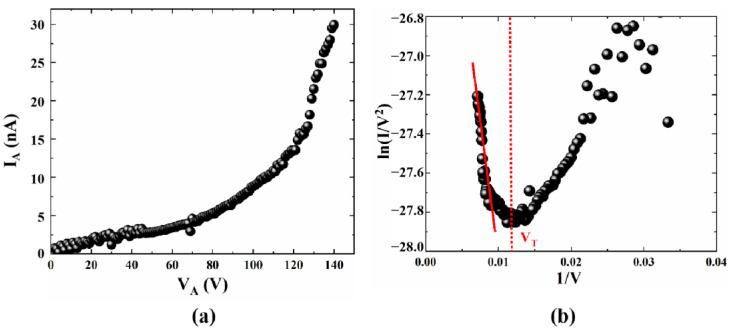
The electrical performance test curves, (**a**) emission current versus collector voltage and (**b**) F-N fitting curve.

To start, we tested the electric performance of the planar structure with no gate bias, as shown in Figure 6a. It is noted that the emission current slowly increases with the initially low collector voltage, and exponentially increases with the rising voltage. On the other hand, the corresponding F-N fitting curve obtained in Figure 6b is based on the transformation of I_A_ and V_A_. An approximately straight line at higher voltages can be clearly observed, which indicates that the emission current follows a typical field emission process. For the F-N fitting curve, the numerical point at which the slope turns from positive to negative can be defined as the turn-on voltage V_T_, which demonstrates the field emission capability of the emitter. It can be seen that the flat emitter structure illustrates a turn-on voltage V_T_ of about 80 V, and the maximum emission current is about 30 nA with the collector voltage further increasing to 140 V; the electric performance is far lower than the reported devices. Therefore, further optimization of the structure parameters is necessarily required to enhance the field emission performance. As shown in Figure 6b, the FN fitting curve shows that the emission efficiency increases with increasing field. The straight line at high electric fields is consistent with classical FN theory; however, the curve bends upwards at low electric fields. FN equations can be applied to solve classical Schrödinger equations to confirm that no nanoscale quantum confinement effects occur. At the nanoscale, electrons are affected by nanoscale quantum effects. The low-energy electrons in the emitter cannot cross the broad vacuum barrier through the FN tunneling when the applied electric field is too weak. The weak current that occurs at this point is mainly due to a spot of electrons ejected from the surface of the emitter by the space charge effect. When the electric field is further increased, the electrons in the emitter escape over the vacuum barrier, which is weakened through the tunneling effect. At this point, the electron emission pattern changes, and the FN theory replaces the space charge effect as the dominant factor, which makes the emission current sharply increase. The point in the curve where the bend occurs can be regarded as the turn-on voltage [27].

### 3.3. Optimization of Back-Gate NVCT

**Figure 7:**
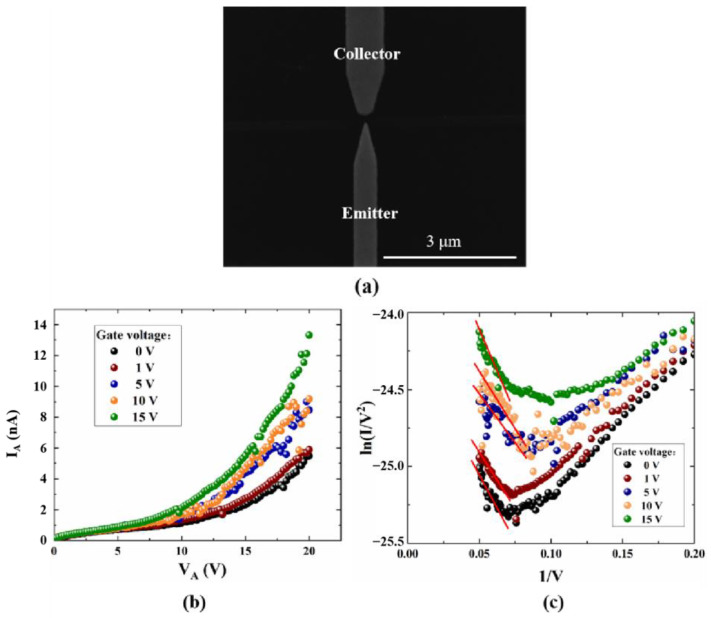
The electrical performance test curves, (**a**) SEM images of back-gate NVCT, (**b**) emission current versus collector voltage, and (**c**) F-N fitting curve.

For conventional solid-state and vacuum transistors, the gate structure is usually realized for modulation, which can enable the device to switch from on-state to off-state. Among the existing reported results, NVCTs are mainly divided into two structural types: back-gate and side-gate, which are compatible with semiconductor processes. On the other hand, the above simulation shows the radius of curvature can be reduced to improve the electric performance, such as the turn-on voltage and the output current. In this case, we further fabricate the back-gate NVCT with a sharp emitter, as is shown in Figure 7a. The shape of the emitter in the simulation is semi-circular, and the emitter is prepared as a sharp cone in the experiments. In practice, it is difficult to fabricate a circular structure, so we choose a sharp cone with a lower curvature than a semicircle.

The back-gate NVCT is fabricated on the SiO_2_/Si substrate, with the silicon oxide acting as the insulating layer, while the doped silicon substrate acts as the back gate. The nanoscale vacuum channel between the emitter and the collector is about 100 nm. According to the tip effect, the smaller the curvature of the cathode tip, the greater the field enhancement factor around it and the consequent increase in field emission current. From the comparison of Figure 6a and Figure 7b, it can be seen that the optimized structure has a collector current of approximately 6 nA at an anode voltage of 20 V when the gate voltages are both zero, and the flat emitter structure requires a collector voltage of 80 V or more to achieve this value. This is consistent with the results obtained from the theoretical analysis. It is observed that optimizing the emitter shape from a flat rectangle to a curved tip can greatly improve device performance, as shown in Figure 7b,c. According to the F-N fitting curve, the turn-on voltage of the device is about 14.8 V, and the corresponding turn-on electric field is about 148 V/μm. Compared to the flat emitter structure, the turn-on voltage has been significantly reduced by optimizing the emitter morphology. In addition, the emission current also significantly increases with the rising gate voltage at a fixed collector voltage. Furthermore, there is an approximately straight line at higher collector voltages from the F-N fitting curve, which indicates that the emission current follows the F-N tunneling mechanism.

### 3.4. Optimization of Side-Gate NVCT

**Figure 8:**
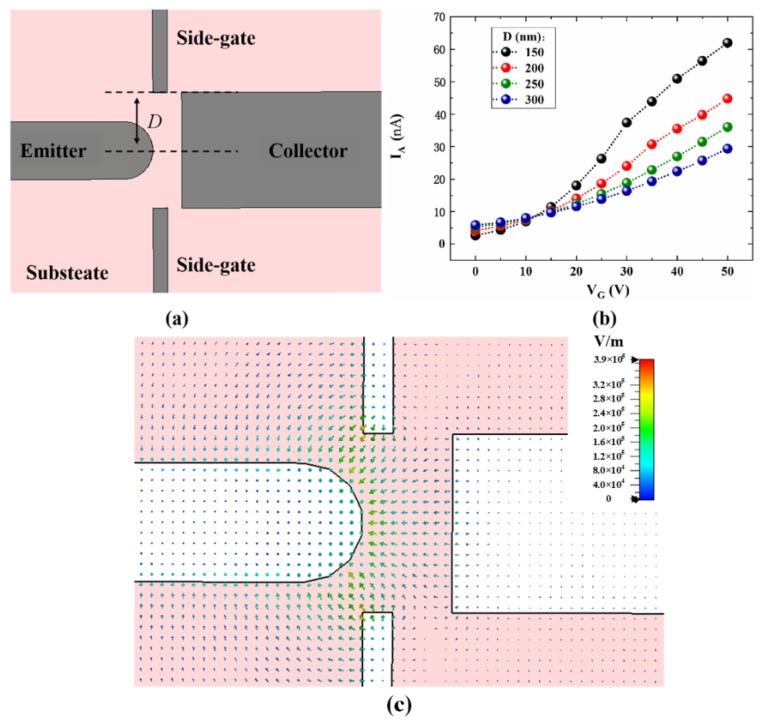
(**a**) top view of structural model of side-gate devices, (**b**) I_A_ versus V_G_ with different distance D between gate to the center line with V_A_ of 20 V, (**c**). electric field of side-gate devices.

Based on the planar-type devices, we further designed a side-gate NVCT, as shown in Figure 8. Figure 8a shows a top view of the simulation model of the side-gate NVCT, with the dual gates located on both sides of the emitter and the collector. The vacuum channel length is set at 100 nm, with the side gate at a distance of D from the center line. The distance between the side-gate and the emitter becomes closer with the decreasing D, such that the modulation effect of the gate becomes more significant. Furthermore, numerical simulations were carried out for the side-gate structure. Figure 8b shows the variation in emission current versus gate voltage for different D values with a fixed collector voltage VA = 20 V. We find that the emission current continuously increases with decreasing D at the same gate voltage. This illustrates the strong modulation of the side-gate, which is consistent with the reported results. On the other hand, the increase in gate voltage not only enhances field emission, but also increases scattering. The emission current increases with increasing V_G_, while the electron utilization of the device is decreasing. Based on the simulation results, we selected 150 nm as the interval between the emitter and the collector.

**Figure 9:**
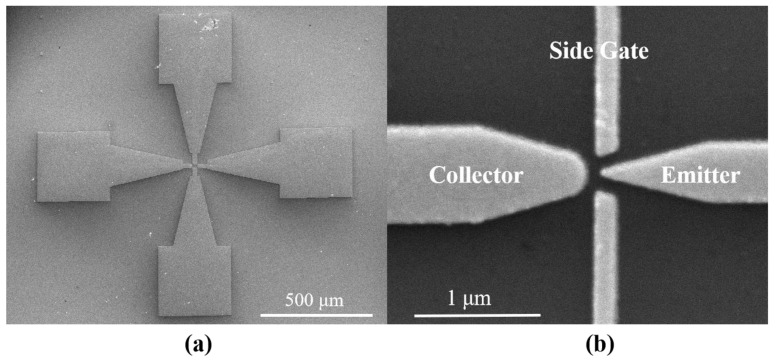
SEM images of (**a**) side-gate NVCT, (**b**) the zoom-in image of the nanoscale vacuum channel.

**Figure 10:**
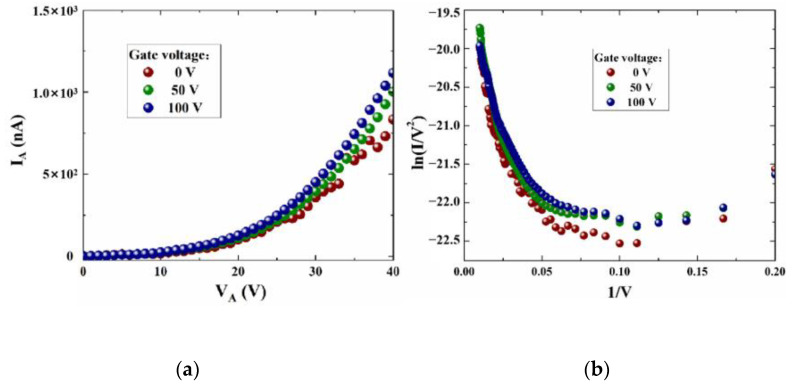
(**a**) I_A_ versus V_A_ at different gate voltages, (**b**) F-N fitting curve.

The shape of the emitter in the simulation is semi-circular, and the emitter is fabricated as a sharp cone in the experiments. On the one hand, it is obvious to find that the emission current increases with the decrease in emitter tip curvature from the simulation results. On the other hand, it is difficult to fabricate a circular structure in practice, so we chose a sharp cone with a higher curvature than a semi-circle. Therefore, we fabricated a side-gate NVCT based on the simulation results. As shown in Figure 9, we can see that the distance from the emitter tip to the collector and from the gate to the emitter is 100 nm and 150 nm, respectively. It is true that the collector and gate do interact with each other due to their small distance. The electric field that causes field emission from the emitter consists of both the gate and the collector, but it is clear that the field is dominated by the gate. Future work will increase the insulation layer to isolate the leakage current and increase the gate distance to reduce the influence between the electrodes. Figure 10 shows the emission current curves at different gate voltages and the corresponding F-N fit curves. It can be seen that the emission current can be improved by the gate voltage, while the modulation effect is not as strong as in the simulation. We assume that this is because of the distance from the gate to the emitter, so the influence of the gate electric field distribution at the emitter tip is insufficient. However, reducing the distance between the gate and the emitter would lead to a large gate leakage current. This so-called trade-off effect should be further considered. Furthermore, according to the F-N fitting curve, the device has a turn-on voltage of approximately 15 V with a corresponding turn-on electric field of about 150 V/μm, which is significantly improved for the flat emitter.

In summary, by optimizing the emitter morphology and gate structure, the turn-on voltage and emission current of the devices are improved. The results show that the structural design of NVCTs still has great potential to be exploited. For instance, attempts to explore patterned emitter array structures could be further considered. During the test of gold nano-vacuum channel structures, the gold electrodes are easily destroyed by evaporation or sublimation, affecting the sustainability of the device. The reason may be that the current density in the test was too large and the heat could not be dissipated in time. This could be addressed by increasing the emissive area of the emitter, such as by increasing the width of the source electrode and designing an array structure to improve it. The field emission performance of the nano vacuum channel structure directly affects the emission current and effective electron utilization of the device. The electron sources tested have low drain currents and an array structure design may be able to increase the emission current to meet the application requirements.

## 4. Conclusions

In this paper, gold nano-vacuum channel structures were fabricated using a high-precision electron beam lithography process. The electrical properties were tested in a vacuum chamber of ~10^−4^ Pa at room temperature. By optimizing the emitter morphology and gate structure, the turn-on voltage and emission current of the devices were improved. During the measurement, the gold electrodes were easily destroyed by evaporation or sublimation, affecting the stability of the device. Due to surface impurities or uneven tips, instantaneous large emission currents may be generated during measurement and destroy the emitters. Therefore, the structural design of NVCT still has great potential to be exploited. For instance, attempts to explore patterned emitter array structures could be further considered, which improves the stability of the emitter. In addition, NVCTs show the potential to realize high integrations of vacuum electron transistors, which further makes it possible to miniaturize vacuum devices, meeting the needs for ultra-fast response times and high-frequency applications.

## Data Availability

We declared that materials described in the manuscript, including all relevant raw data, will be freely available to any scientist wishing to use them for noncommercial purposes, without breaching participant confidentiality.
